# Impact of an Irrigation Dam on the Transmission and Diversity of *Plasmodium falciparum* in a Seasonal Malaria Transmission Area of Northern Ghana

**DOI:** 10.1155/2020/1386587

**Published:** 2020-03-19

**Authors:** Eric Kyei-Baafour, Bernard Tornyigah, Benjamin Buade, Langbong Bimi, Abraham R. Oduro, Kwadwo A. Koram, Ben A. Gyan, Kwadwo A. Kusi

**Affiliations:** ^1^Department of Immunology, Noguchi Memorial Institute for Medical Research, College of Health Sciences, University of Ghana, Legon, Ghana; ^2^Department of Animal Biology and Conservation Science, College of Basic and Applied Sciences, University of Ghana, Legon, Ghana; ^3^Navrongo Health Research Centre, Ghana Health Service, Navrongo, Ghana; ^4^Department of Epidemiology, Noguchi Memorial Institute for Medical Research, College of Health Sciences, University of Ghana, Legon, Ghana

## Abstract

Water bodies such as dams are known to alter the local transmission patterns of a number of infectious diseases, especially those transmitted by insects and other arthropod vectors. The impact of an irrigation dam on submicroscopic asexual parasite carriage in individuals living in a seasonal malaria transmission area of northern Ghana was investigated. A total of 288 archived DNA samples from two cross-sectional surveys in two communities in the Bongo District of Northern Ghana were analysed. Parasite density was determined by light microscopy and PCR, and parasite diversity was assessed by genotyping of the polymorphic *Plasmodium falciparum* msp2 block-3 region. Submicroscopic parasitaemia was estimated as the proportional difference between positive samples identified by PCR and microscopy. Dry season submicroscopic parasite prevalence was significantly higher (71.0%, *p*=0.013) at the dam site compared with the nondam site (49.2%). Similarly, wet season submicroscopic parasite prevalence was significantly higher at the dam site (54.5%, *p*=0.008) compared with the nondam site (33.0%). There was no difference in parasite density between sites in the dry season (*p*=0.90) and in the wet season (*p*=0.85). Multiplicity of infection (MOI) based on PCR data was significantly higher at the dam site compared with the nondam site during the dry season (*p* < 0.0001) but similar between sites during the wet season. MOI at the nondam site was significantly higher in the wet season than in the dry season (2.49, 1.26, *p* < 0.0001) but similar between seasons at the dam site. Multivariate analysis showed higher odds of carrying submicroscopic parasites at the dam site in both dry season (OR = 7.46, 95% CI = 3.07–18.15) and in wet season (OR = 1.73, 95% CI = 1.04–2.86). The study findings suggest that large water bodies impact year-round carriage of submicroscopic parasites and sustain *Plasmodium* transmission.

## 1. Introduction

The global malaria burden over the past decade has declined, though sub-Saharan Africa continues to suffer most from the disease, contributing over 88% of the global burden [1]. As such, policy makers have prioritized several interventions such as the use of LLINs, use of RDTs to confirm cases prior to treatment, seasonal chemoprevention for children, and IPTp for pregnant women over the past decade to further reduce and prevent a surge in disease burden [2].

While these have achieved some degree of success, carriage of submicroscopic parasites by some individuals within endemic communities still poses a threat as such individuals serve as reservoirs of infection [3]. Individuals with submicroscopic parasites are estimated to transmit between 20% and 50% of almost all human-to-mosquito infections [4]. As the disease burden declines with low transmission, infections tend to cluster around areas with ecologically suitable sites for vector survival [5].

Areas around dams and large fresh water bodies for both irrigation and hydroelectric power generation are potential “hotspots” for transmission of the disease [6]. This is evident in the prevalence of vector-borne diseases such as malaria [7], schistosomiasis [8], and other water-borne infections such as diarrhea, Guinea worm, and lymphatic filariasis around such sites especially in tropical areas. For malaria, infected persons in these areas may not necessarily show disease symptoms, and carriage of submicroscopic parasites will perpetuate transmission even during generally dry periods when transmission is expected to be very low or null. There is ample evidence showing that submicroscopic levels of asexual malaria parasites are enough to generate gametocytes to maintain effective disease transmission [4, 9]. Asymptomatic individuals including those with very low, submicroscopic infections are therefore an important group for the malaria elimination agenda as they can be efficient reservoirs for transmission in the presence of vectors [3].

Genetic diversity of *P. falciparum* has been associated with evolutionary fitness, and multiple strain infections have been demonstrated in areas of high transmission [10, 11]. Some of these diverse parasites may be drug-resistant strains [12], and there is therefore a need to study effect of the dam's presence on the diversity of malaria parasites in the study area.

Although large dams improve the socioeconomic status of inhabitants, the health challenges that come with such projects need to be addressed. Therefore, clearer understanding of how large artificial water bodies impact on submicroscopic *Plasmodium* carriage, which can potentially form an essential transmission reservoir, can shape how both active and passive surveillance systems are pursued in the quest to eradicate malaria. This study therefore investigated the effect of an irrigation dam on the carriage of submicroscopic parasitaemia by comparing individuals living around the dam with those who are at least 20 km away from the dam.

## 2. Materials and Methods

### 2.1. Study Area and Study Population

This study was a nested study carved out from a larger cross-sectional community survey. The original study was conducted in two communities in the Bongo District in the Upper East Region of Ghana; the Gowrie/Vea community which has the Vea irrigation dam (dam site) and the Soe community, about 20 km away (nondam site). The study area and population have been described in detail previously [13, 14]. Briefly, malaria transmission in Northern Ghana is perennial but seasonal, with marked high transmission during the rainy or wet season and low transmission in the dry season [15, 16]. The wet season begins in June and ends in October while the dry season spans between the months of November and May. It is estimated that the annual *P. falciparum* entomological inoculation rate (EIR) in the district is about 25.3 infectious bites per person per year [17] with malaria attack rate being 3.5/child in a year [14]. Stored filter paper blots sampled from individuals recruited at the end of the wet season in November/December 2012 and from a similar but independent survey conducted at the end of the dry season in April 2013 were used for this study. Thick and thin smears were made for the determination of parasitaemia. A total of 288 filter paper samples (154 and 134 for wet and dry seasons, respectively) from participants aged one to seventy years were analysed in this study.

### 2.2. Laboratory Analysis

#### 2.2.1. Examination of Blood Smears

For the original study, thick and thin blood smears were examined by an experienced microscopist using a light microscope. About 100 high-powered fields (using oil immersion at 100X magnification) of the thick film were examined before a slide was deemed negative for *Plasmodium*. Parasite density was determined by counting the number of parasites against 500 white blood cells (WBCs). Number of parasite/*μ*l was determined using a standard assumed leucocyte count of 8,000/*μ*l.

#### 2.2.2. DNA Extraction and *P. falciparum* Detection by PCR

DNA extraction was done using the Chelex method [18, 19]. In brief, each blotted filter paper was cut into a 1.5 ml Eppendorf tube and 1 ml of PBS (pH 7.4) and 50 *μ*l of 10% saponin were added and incubated overnight at 4°C. The tubes were centrifuged at 14,000 rpm for 30 seconds, and the supernatant was discarded. One millilitre (1 ml) of PBS was added to the tube and incubated for 30 minutes at 4°C. The tube containing the solution was centrifuged for 2 minutes, and the supernatant was discarded. About 100 *μ*l of sterile distilled water was added to each tube followed by the addition of 50 *μ*l of 20% Chelex solution. The tubes were vigorously vortexed and incubated at 95°C for 10 minutes and vortexed 5X at 2-minute intervals. The tubes were then centrifuged at 13,000 rpm for 5 minutes, and the supernatant DNA was stored at −20°C.

Polymerase chain reaction (PCR) was used in the detection of *P. falciparum* among the isolates using a previously described protocol with some modifications [20]. In brief, detection of *Plasmodium* was done using the genus-specific primers (rPLU 5 and rPLU6) which target the genes of small subunit ribosomal RNA (ssrRNA) sequences. The reaction had 4 mM of MgCl_2_, 200 *μ*M DNTPs, 0.0625 *μ*M of each primer, and a unit of Taq DNA polymerase (Sigma-Aldrich, St Louis, MO, USA) with 5 *μ*l DNA. Cycling conditions were as follows: initial denaturation at 94°C for 15 minutes preceded 32 amplification cycles: denaturation at 94°C for 1 minute and annealing at 58°C for 2 minutes. Extension was at 72°C for 2 minutes and final extension at 72°C for 10 minutes. All the reactions were carried out using the S1000 thermal cycler (Bio-Rad Inc., USA).

#### 2.2.3. Genotyping of *P. falciparum* msp2

Genotyping of DNA isolates was done by the amplification of the polymorphic block-3 region of the *msp2* gene using a PCR protocol by Snounou et al. [21] with slight modification. Primary or outer primers corresponded to conserved sequences flanking the repetitive regions (Table 1), and the secondary or nested primers amplified the 3D7 and FC27 allelic families. All reactions had genomic DNA from 3D7 and HB3 laboratory strains as positive controls and molecular grade water as the negative control. The nested PCR products were analysed by electrophoresis on 2% agarose gel stained with ethidium bromide. The DNA (bands) was visualized using UV transillumination, and fragments obtained were scored with reference to a standard 100 bp ladder loaded onto the gel. Positive samples were recorded with their band size and the number of bands per sample, which refers to the clones in a sample. Samples with single bands were considered mono-infections and those with two or more bands considered multiple infections.

### 2.3. Data Analysis

Chi-square/Fisher's exact tests were used to compare differences in proportion of individuals carrying parasites between sites and across seasons. Multiplicity of infection was calculated by determining the mean number of parasite clones in each cohort. Relationship between dam presence and submicroscopic parasite carriage was assessed using a multiple logistic regression model adjusting for sex, bednet use, and age as potential confounders. Submicroscopic parasitaemia is defined as individuals whose infections were detected by PCR only and not by microscopy. Analyses were performed using the R statistical package (R version 3.4.2, 2017). For all analyses, differences with a *p* value equal to or less than 0.05 were deemed to be statistically significant.

## 3. Results and Discussion

### 3.1. Study Participants

A total of 288 filter paper samples were analysed in this study, with a breakdown as follows: 154 samples from the wet season included 66 samples from the dam site and 88 samples from the nondam site. 134 samples from the dry season included 69 samples from the dam site and 65 from the nondam site. Mean age at the dam site was 17.8 years compared with 16.9 years at the nondam site (*p*=0.61) (Table 2).

### 3.2. Asexual *Plasmodium falciparum* Carriage

**Figure 1:**
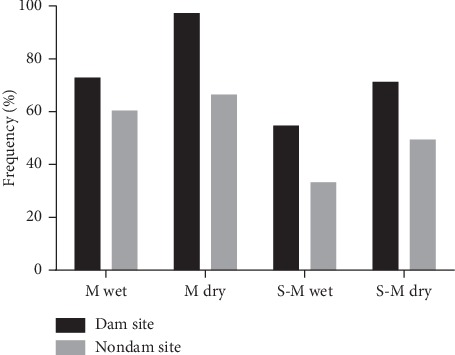
Parasite prevalence by microscopy and PCR. Frequency distribution of the prevalence of *P. falciparum* by microscopy and PCR at both sites in the wet and dry season. Labels for the *x*-axis: M wet-wet season microscopy, M dry-dry season microscopy, S-M wet-wet season submicroscopic, and S-M dry-dry season submicroscopic.

At the dam site, wet season parasite prevalence was 18.2% by microscopy and 72.7% by PCR, while dry season parasite prevalence was 26.1% by microscopy and 97.1% by PCR (Figure 1). Wet season parasite prevalence at the nondam site was 27.3% by microscopy and 60.2% by PCR, while the dry season parasite prevalence was 26.1% by microscopy and 66.2% by PCR (Figure 1). There were no significant differences in parasite prevalence by microscopy between the two study sites in both wet and dry seasons (*p* > 0.05 in both cases, 2-sample test for equality of proportions). Parasite prevalence rates by PCR were significantly different between the sites during the dry season (*p* < 0.001) but not during the wet season (*p*=0.12). All microscopy-positive isolates were also positive by PCR. Mean parasite density from microscopy in the wet season at the dam site was 468 parasites/*μ*l and 399 parasites/*μ*l at the nondam site. In the dry season, parasite density was 318 parasites/*μ*l at the dam site and 423 parasites/*μ*l at the nondam site. Parasite density did not differ between the dam and nondam sites in either season.

### 3.3. Submicroscopic Infection Estimation

Submicroscopic infection, determined as the difference in parasite prevalence detected by PCR and microscopy, was 54.5% at the dam site and 33.0% at the nondam site in the wet season. In the dry season, it was 71.0% at the dam site and 49.2% at the nondam site. The prevalence of submicroscopic infection was consistently higher at the dam site relative to the nondam site in both wet (*p*=0.008) and dry (*p*=0.013) seasons (Fisher's exact test for proportional differences) (Figure 1). Submicroscopic infection in the dry season was 82.1% compared with the wet season 65.6% (*p*=0.002).

### 3.4. Parasite Diversity and Multiplicity of Infection

Nine different 3D7 alleles were detected at the dam site and 12 alleles at the nondam site in the wet season (band sizes ranging from 200 bp to 700 bp for both sites). For FC27 alleles, 8 (from 250 bp to 500 bp) were detected at the dam site and 12 (200 bp–600 bp) from the nondam site. The predominant allele for 3D7 at both sites in the wet season is 300 bp and that of FC27 is 400 bp.

**Figure 2:**
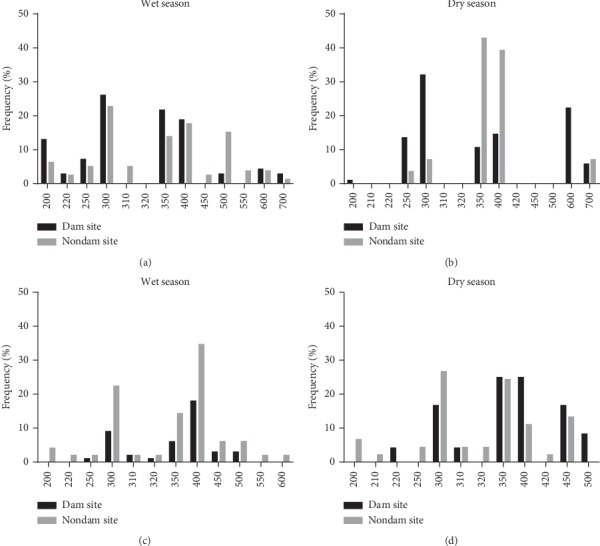
Allelic distribution of the two families of the *msp2* gene. Allelic frequency of msp2 families. (a) and (b) represent the frequency of 3D7 alleles between sites while (c) and (d) represent that of FC27.

In the dry season, seven 3D7 (200 bp–700 bp) and seven FC27 (220 bp–500 bp) alleles were detected at the dam site while five 3D7 (250 bp–700 bp) and 10 FC27 (200 bp–450 bp) alleles were detected at the nondam site (Figure 2). In the dry season, 300 bp was the predominant allele for 3D7 at the dam site while 350 bp was predominant at the nondam site. The nondam site in the dry season had the lowest number of individuals carrying both allelic families. These notwithstanding, the proportions of individuals with the 3D7 and FC27 allelic forms of the parasite were similar between the dam and nondam sites.

Multiplicity of infection (MOI), determined as the number of concurrent *P. falciparum* clones infecting an individual at a particular time, was estimated and compared between the dam site and the nondam site and across seasons (Table 3). MOI was significantly higher at the dam site (2.24) compared with the nondam site (1.26) in the dry season (*p* < 0.0001) but similar between sites in the wet season (dam = 2.42, nondam = 2.49, *p*=0.78, Table 3). MOI was also significantly higher in the wet season (2.49) compared with the dry season (1.26) at the nondam site (*p*=0.0002) but similar between seasons at the dam site (Table 3).

The proportions of individuals with single allele infections and multiple allele infections, based on the msp2 typing data, were compared between the two study sites. There was a higher proportion of multiple allele infections at the dam site and a higher proportion of single allele infections at the nondam site during the dry season (*p* < 0.0001, Fisher's exact test, Table 3). In the wet season, however, the proportions of single and multiple allele infections were similar at the two sites (*p*=0.83).

### 3.5. Factors Predicting Submicroscopic Parasite Carriage

Factors predicting submicroscopic *P. falciparum* carriage were separately analysed for the wet and dry seasons. Multiple logistic regression analysis was performed with parasitaemia as a binary outcome variable and with the study site as the predictor variable. Models were corrected for bednet usage as a confounder since there were significant differences in usage between the two study sites (Table 4). The dam site was significantly associated with increased odds of submicroscopic parasite carriage in both wet (OR 1.73; 95% CI = 1.04–2.86) and dry (OR 7.46; 95% CI = 3.07–18.15), *p* < 0.0001) seasons.

## 4. Discussion

Malaria is an important infectious disease whose transmission could be sustained by the presence of vector-breeding sites such as dams. This study therefore sought to evaluate the impact of an irrigation dam on the carriage of submicroscopic parasites in an area of seasonal malaria transmission in Northern Ghana.

The study data generally show a significant effect of the dam on submicroscopic parasite prevalence in this seasonal malaria transmission area, especially in the dry season. Parasite prevalence as detected by PCR was proportionally higher in all instances compared with prevalence detected by microscopy. Submicroscopic parasite levels were significantly higher at the dam site during the dry season (Figure 1). Similar findings of high prevalence of submicroscopic parasites have been reported in some communities in Ghana [22]. These observations could be related to the presence of large water bodies in these communities, and this provides direct evidence of the impact of the dam on the persistence of parasites for sustained transmission during this period. Generally high prevalence of submicroscopic *P. falciparum* parasites during both wet and dry seasons at the two study sites indicates the potential for transmission to occur throughout the year or at least have parasites persist throughout the dry season and be ready for transmission when vector breeding commences in the wet season. Currently, in Northern Ghana, there is an on-going seasonal malaria control programme that administers antimalarial drugs during the wet season only [23] in an effort to reduce parasite burden. The assumption is that very limited or no transmission takes place during the dry season. This strategy therefore does not account for the presence of large water bodies such as dams, which may not completely dry up during these periods. Moreover, the Ghana Government, through its one-village-one-dam policy [24], aims to create additional dams especially in the northern part of the country to sustain agriculture and livestock breeding during the dry season. The seasonal malaria control strategy focuses on chemotherapy during the wet season [23, 25], and the current data, in agreement with a previous finding [22], identify a potential loophole in this control strategy, especially in communities that have very large dams. Our data therefore highlight the need to factor the presence of such water bodies into malaria control efforts.

Studies in the same region have shown a high human-biting rate in general with the *S*. form of *An. gambiae s.l.* being the predominant parasite vector around irrigated areas [16]. Vector population in these areas has been reported to be high even during the dry seasons [26], and although malaria transmission has been described as seasonal in these areas, transmission around the dam site may not be seasonal but rather occur throughout the year. We did not determine the extent of submicroscopic gametocyte carriage in this study, but untreated asexual submicroscopic parasites have been shown to potentially develop into gametocytes for onward transmission into human hosts [27].

*Plasmodium falciparum* diversity was investigated by molecular typing of the polymorphic merozoite surface protein 2 (MSP-2) block-3 region which is often used as a marker to study diversity of parasite populations [28–30]. The study showed a moderate-to-high *P. falciparum* allelic diversity with the 3D7 allelic family being the most predominant variant circulating in the study communities (Figure 2). This is similar to data reported from other regions of Ghana and countries in sub-Saharan Africa with similar malaria transmission settings [29, 31–33]. A study conducted in the south of Benin however reported the FC27 family as the most predominant allelic family [34]. The reported differences in the genetic diversity of *P. falciparum* infections may be due to host-parasite interactions, transmission intensity, ethnicity, and geographical location.

Multiplicity of infection (MOI), which is defined as the mean number of *P*. *falciparum* genotypes an individual is infected with, was also investigated. MOI was significantly higher at the dam site compared with the nondam site in the dry season (*p* < 0.0001) but similar between sites in the wet season (*p*=0.78) (Table 3). MOI was also found to be significantly higher in the wet season compared with the dry season at the nondam site (*p*=0.0002) but not at the dam site (Table 3). These data agree with the report by Duah et al. [33] who found similar MOI in the Savannah regions of Ghana. Although this study did not directly assess the levels of gametocytes and malaria transmission by the vector, there are published reports of a direct correlation between MOI and transmission intensity [35], and the higher dam site MOI could mean a higher extent of transmission at this site during the dry season compared with the nondam site. This is therefore indicative of the potential of equally high transmission at the dam site in the dry season though the area has been described as having seasonal transmission [16]. Similar levels of single and multiple parasite variant infections were observed at both study sites during the wet season, but a high number of multiple variants were detected at the dam site compared with the nondam site during the dry season (Table 3). The observation at the dam site in the dry season further corroborates the potential of higher disease transmission at this site during the dry season.

The relationship between submicroscopic infection and presence of the dam was investigated using multivariate models after adjusting for sex, age, and bednet usage (Table 4). There was a strong significant association of the dam with submicroscopic *P. falciparum* carriage in both wet and dry seasons, and the odds were even much higher in the dry season compared with the wet season. These further corroborate the impact of the dam on the persistence of *Plasmodium* infections during the dry season and hence malaria transmission. Similar findings of increased risk of malaria within irrigated areas elsewhere in Africa have been reported previously [36–38]. Construction of dams for irrigation and hydroelectricity to promote socioeconomic development in Africa has been projected to increase [39], but persistent malaria transmission could be an unintended consequence in these communities if adequate control measures are not implemented. These findings collectively suggest a re-examination of malaria case detection and a possible rethink of seasonal malaria chemoprevention strategies in such communities. This is also important because as malaria transmission decreases due to effective control strategies, the parasite threshold that can result in clinical malaria symptoms is likely to reduce and parasite densities that are currently considered asymptomatic may become clinically relevant [40]. High burden of submicroscopic infection if not treated could influence diversity of circulating strains in the study area which may intend alter the transmission dynamics in the study communities. Though the study did not directly assess entomological inoculation rate (EIR), reports from different malaria transmission areas have described high human-biting rate around irrigated areas [37, 41], and this could pertain to our study site. Also, the use of seasonal chemoprevention in these areas will most likely reduce the tendency that parasites detected in the dry season are a possible carry over from the rainy season.

## 5. Conclusions

The presented data highlight the impact of irrigation dams on the diversity and transmission dynamics of *Plasmodium falciparum* in Northern Ghana. Following the call for construction of additional dams in these areas for economic development, it is important to holistically reflect on the potential health impact of these dams on transmission of diseases such as malaria.

## Figures and Tables

**Table 1:** Primer sequence for amplifying *msp2* gene of *Plasmodium falciparum*.

Primer	Sequence
Primary/outer Fw	5′-ATGAAGGTAATTAAAACATTGTCTATTATA-3′
Primary/outer Rv	5′-CTTTGTTACCATCGGTACATTCTT-3′
3D7 Fw	5′-GCT TAT AAT ATG AGT ATA AGG AGA A-3′
3D7 Rv	5'-CTG AAG AGG TAC TGG TAG A-3′
FC27 Fw	5′-GCT TAT AAT ATG AGT ATA AGG AGA A-3′
FC27 Rv	5′-GCATTGCCAGAACTTGAA-3′

**Table 2:** Population characteristics of selected samples.

Covariate		Gowrie/Vea (dam site) (*n* = 135) (%)	Soe (nondam site) (*n* = 153) (%)	Total (*n* = 288) (%)	*p* value
Season					
Dry		69 (51.1)	65 (42.5)	134 (46.5)	
Wet		66 (48.9)	88 (57.5)	154 (53.5)	0.18^*∗*^
Mean age (sd)		17.8 (13.3)	16.9 (15.1)	17.3 (14.3)	0.61^#^

Bednet				
No		46 (34.1)	28 (18.3)	74 (25.7)	
Yes		89 (65.9)	125 (81.7)	214 (74.3)	0.003^*∗*^

Sex				
F		67 (49.6)	89 (58.2)	156 (54.2)	
M		68 (50.4)	64 (41.8)	132 (45.8)	0.18^*∗*^

^#^Differences in mean age between communities were determined using Student's *t*-test. ^*∗*^Proportional differences between communities for season, sex, and bednet usage were determined using Chi-square test. (%) Numbers in brackets are percentages.

**Table 3:** Multiplicity of infection estimates using *msp2* loci and proportions of individuals carrying multiple and single clones.

	Season	Infection	Dam site (%)	Nondam site (%)	*p* value
Clones	Wet	Single	16 (33.3)	16 (30.2)	0.831^*∗*^
		Multiple	32 (66.7)	37 (69.8)	
	Dry	Single	18 (26.9)	34 (79.1)	<0.001^*∗*^
		Multiple	49 (73.1)	9 (20.9)	

MOI	Wet		2.42	2.49	0.778^≠^
	Dry		2.24	1.26	<0.001^≠^
	*p* value^*β*^		*0.413*	<0.001	

^*∗*^ *p* values obtained using Fisher's exact test for proportions. *p* values obtained following MOI comparison between seasons (^*β*^) or between sites (^≠^) using Mann–Whitney test.

**Table 4:** Predicting submicroscopic parasitaemia based on dam presence.

Season		Unadjusted		Adjusted	
		Odds ratio	95% CI	Odds ratio	95% CI
Wet	Microscopic^*∗*^	1.41	(0.93–2.15)	1.40	(0.89–2.73)
	Submicroscopic^#^	1.73	(1.09–2.75)	1.73	(1.04–2.86)
Dry	Microscopic^*∗*^	6.59	(3.60–12.08)	8.03	(4.16–15.51)
	Submicroscopic^#^	4.60	(2.25–9.83)	7.46	(3.07–18.15)

^*∗*^Isolates positive by microscopy only. ^#^Isolates positive by PCR only but negative by microscopy.

## Data Availability

The data used to support the findings of this study are included within the article.
